# Link between human ABO blood groups with diseases influencing blood donors and recipients frequency at RBTC, Delhi, India

**DOI:** 10.6026/97320630019576

**Published:** 2023-05-31

**Authors:** Sanjay Kumar Thakur, Singh Sompal, Negi Dinesh Kumar, Anil Kumar Sinha

**Affiliations:** 1 PG Department of Zoology, Veer Kunwar Singh University, Ara, Bihar - 802301, India; 2 Department of Regional Blood Transfusion Centre, Hindu Rao Hospital and NDMC Medical College, Delhi – 110007, India

**Keywords:** Blood group, Blood donor, Blood component, Blood component recipients, ABO and Rh blood group

## Abstract

Blood groups had associations with many diseases that affect blood transfusion services by increasing or decreasing the blood demand of particular blood group. The present study was designed to compare the frequency of ABO and Rh blood groups among blood donors and blood component recipients. The ABO and Rh(D) blood groups of donors and recipients were determined using Gel card method. The frequency of blood donors and blood component recipients from January 1, 2020, to December 31, 2023, at regional blood transfusion centre of Delhi, were compared using χ² test. The ABO blood group frequencies of blood donors (n=23025) were: A(23.1%), B(37.53%), AB(10.09%), and O(29.29%). The blood issue (n=20255) was significantly (p=0.0000) higher in A(24.96%), B(39.92%), and lower in AB(9.76%) and O(25.37%). The RDP issue (n=7239) was significantly (p=0.0000) higher in A(24.71%), B(39.34%), and AB(11.53%) and lower in O(24.41%). The FFP issue (n=4164) was significantly (p=0.00024) higher in AB (12.3%) and lower in A (22.05%), B(37.32%), and O(28.14%). The difference between the blood donor frequencies of Rh(D)+Ve(95.19%) and Rh(D)-Ve(4.81%) and the blood issued by Rh(D)+Ve(95.06%) and Rh(D)-Ve(4.94%) was statistically not significant(P=0.52).Blood issues were higher in blood group A and B than in O, platelet issues were higher in A, B and AB than in O, and FFP issues were higher in the AB. Non-O blood groups may have a higher frequency of blood transfusions, while O blood groups may have a protective influence against diseases due to their innate immune response.

## Background:

Karl Landsteiner discovery of the ABO blood group system in 1900 [1] paved the way for blood transfusion and a wide variety of findings in immunohematology. In 1930, he was awarded the Nobel Prize for this achievement. Alfred Von Decastello and Adriano Sturli discovered the fourth kind of blood group, AB, in 1902[2]. The Rh (Rhesus factor) system was later defined jointly by Landsteiner and Weiner in 1940 [13]. The ABO gene, which encodes the ABO antigen, is located on the long arm of the ninth human chromosome (9q34.1) [4], whereas the Rh(D) gene, which encodes the Rh protein, is located on chromosome 1p34-p36[5]. According to the International Society of Blood Transfusion (ISBT), until December 2022, there will be 44 recognized blood group systems with 354 red blood cell antigens. The 44 systems are defined genetically by 49 genes. Clinically, the ABO and Rhesus (Rh) blood type systems are the most significant [6].

In the body, blood carries nutrition, hormones, metabolic waste products, oxygen, and more. Blood transfusions are necessary in the event of a blood shortage in the body to ensure survival. Blood group antigens are essential for safe and compatible blood transfusion. Blood group antigens are hereditary traits that indicate people's polymorphic features and are found on the plasma membrane of red blood cells (RBCs). Proteins and carbohydrates bound to lipids or proteins in the plasma membrane of red blood cells serve as the ABO and Rhesus Rh blood types defining antigens. Based on the presence of antigens (agglutinogens) on the surface of red blood cells and naturally occurring corresponding antibodies (agglutinins) in their plasma, people are divided into the four main ABO blood groups: A, B, O, and AB. Rh(D) antigen determines positive blood type in the Rh system, whereas its absence determines Rh negative blood group. The antibodies for Rh will develop as a result of an immunological response, and these antibodies might result in hemolysis in vivo following the transfusion of incompatible blood [7].

Blood group antigens are reflections of people's polymorphic traits. The study reports of many authors show that variations in blood type antigen expression may increase or decrease the host's vulnerability to a variety of diseases. Many researchers have linked blood group antigens to a variety of disorders [8]. The association between different blood groups and certain human infections poses health risks, and it has an impact on blood transfusion services by increasing or decreasing blood transfusions of the respective blood group compared to the general population. The frequency of ABO and Rh blood groups in blood donors represents the blood group frequency of the general population of the geographical region.

The frequencies of ABO and Rh blood groups vary greatly among races, geographical borders, and ethnicities; even within the same location. Knowledge of the blood group frequency distribution of the population of blood donors and blood component receivers is necessary for blood transfusion services and to improve blood component inventory management. It is equally important for physicians, patients, and other people. This can minimize patient mortality and morbidity. It aids in the understanding of certain deficient blood groups in a specific location, which aids in deciding how to mobilize volunteer blood donors and urge donors from deficient blood groups to give more regularly. This is important for health planners when developing preventative strategies in a specific region to address future health concerns. This is also important and useful for biological researchers studying inheritance patterns, population genetics, population migration patterns, disease susceptibility, forensic studies, and geographic information for population anthropology. There is no data on the prevalence of ABO and Rh blood groups among blood donors and blood component recipients. Therefore, it is of interest to examine and compare the frequency of ABO and Rh(D) blood groups among blood donors and blood component recipients.

##  Material and Methods:

## Ethics approval and consent to participate:

The institutional ethical review committees of Hindu Rao Hospital and NDMC Medical College, Delhi, approved the present study with permission number F.No. IEC/NDMC/2021/69. All of the participating blood donors gave their consent to donate blood. Only data from the routine blood grouping of blood donors and blood component recipients from blood bank inventory registers was used for the analysis of outcomes in the present study. Since no separate blood sample was requested from volunteers for the present study, no separate informed consent was obtained.

## Study Area and Design:

The present study was conducted at the Regional Blood Transfusion Centre in Delhi, India. The ABO and Rh(D) blood types of blood donors and blood component recipients were studied from January 1, 2020, through December 31, 2023.

## Inclusion criteria:

Only those blood donors who were in good physical health, between the age groups of 18 and 65 years, weighed more than 45 kg., had hemoglobin levels above 12.5 g/dL, and qualified for blood donation as per the standard operating procedure (SOP) of the blood bank, donated blood. Donors who donated blood and patients who receive blood components were included in the study.

## Exclusion criteria:

Donors who were not qualified for blood donation and patients who did not receive blood were excluded from this study.

## Sampling Technique and Laboratory investigations:

All the samples of blood donors and recipients of blood components were collected in EDTA and plain tubes and subjected to ABO and Rh(D) blood type testing. The ABO and Rh(D) blood groups were identified using commercial Gel cards (DiaClon ABO/D+Reverse Grouping, BIO-RAD, Switzerland) and the hem-agglutination technique. The blood grouping test was carried out in accordance with the manufacturer's recommendations.

## Statistical analysis:

Data from the study were gathered and recorded into a Microsoft Excel spreadsheet, and statistical analysis was carried out using the free and open-source statistical programmer R. Descriptive statistics and frequency distribution were analyzed. To display the results, a table and pie chart were used. The Pearson Chi-Square test was used to compare the blood group frequencies of blood donors and recipients. Statistical significance was defined as p-values less than 0.05.

## Result 

Of a total of 23021 blood donors, 22816 (99.11%) were male and 205 (0.89%) were female. The age of male donors (31.16±8.43 years) and female donors (33.33±9.50) had no statistically significant (p=0.108) difference. The hemoglobin levels of male donors (14.58±1.58 gm/dl) and female donors (13.44 ± 0.85 gm/dl) had significant (p = 0.000) differences. The weights of male donors (74.29 ± 11.73 kg) and female donors (65.54 ± 8.01 kg) had significant (p = 0.00) differences.

## Difference between the ABO and Rh (D) blood group frequencies of the blood donor and the blood issued to the recipient:

**Figure 1:**
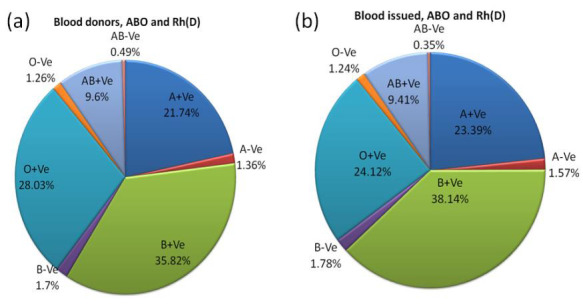
Frequency of ABO and Rh (D) blood groups of blood donors (a) and blood issued (b).

A total of 23021 blood donors were tested for ABO and Rh(D) blood groups in which the frequency of blood group(Figure 1(a)) are as follows; A+Ve, A-Ve, B+Ve, B-Ve, O+Ve, O-VeAB+Ve, and AB-Ve, were 5004 (21.74%), 313 (1.36%), 8247 (35.82%), 392 (1.7%), 6453 (28.03%), 289 (1.26%), 2210 (9.6%), and 113 (0.49%), respectively. A total of 20255 blood units (whole blood and packed red blood cells) were issued to the recipients, of which A+Ve, A-Ve, B+Ve, B-Ve, O+Ve, O-VeAB+Ve, and AB-Ve, were 4738 (23.39%), 318 (1.57%), 7725 (38.14%), 360 (1.78%), 4886 (24.12%), 252 (1.24%), 1905 (9.41%), and 71 (0.35%) respectively(Figure 1(b)). A higher frequency of A+Ve, A-Ve, B+Ve, and B-Ve and a lower frequency of AB+Ve, AB-Ve, O+Ve, and O-Ve of blood issued to the recipient were observed (Figure 1(a) & Figure 1(b)). The difference in ABO and Rh(D) blood group frequency of blood donors and blood issues was statistically significant (χ² =100.622; p = 0.0000).

**Figure 2:**
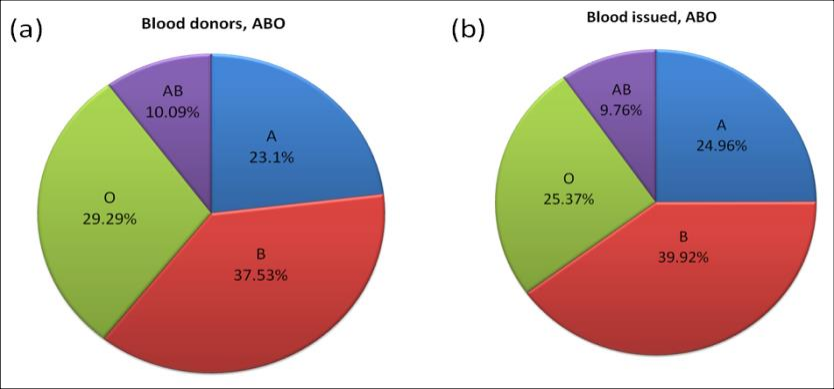
Frequency of ABO blood groups of blood donors (a) and blood issued (b).

**Figure 3:**
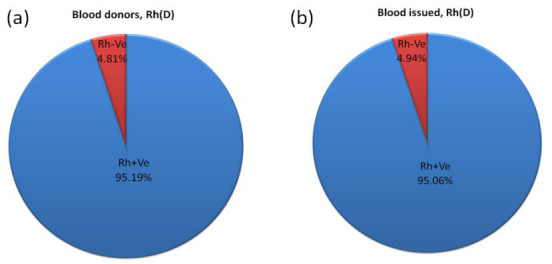
Frequency of Rh blood group of blood donors (c) and blood issued (d).

The A, B, AB, and O blood group frequencies of blood donors (Figure 2(a)) were 5317 (23.1%), 8639 (37.53%), 2323 (10.09%), and 6742 (29.29%), respectively. Compared to the ABO blood group frequency of blood donors, a significantly (p = 0.0000) higher frequency of blood issues (Figure 2(b)) was observed for blood groups A (24.96%) and B (39.92%) and a lower frequency for AB (9.76%) and O (25.37%). The Rh(D)+Ve and Rh(D)-Ve blood group frequencies of blood donors (Figure 3(a)) were 21914 (95.19%) and 1107 (4.81%), respectively, and blood issued (Figure 3(b)) were 19254 (95.06%) and 1001 (4.94%). The difference in Rh(D)+Ve and Rh(D)-Ve blood group frequencies of blood donors and blood issues was statistically not significant (χ² =0.4134018; p = 0.52).

**Figure 4:**
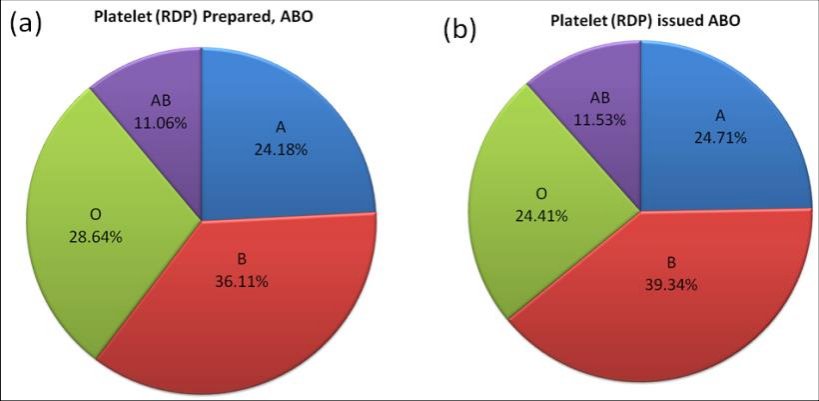
Frequency of ABO blood groups of platelets (RDP) units prepared (a) and platelets (RDP) units issued (b).

**Figure 5:**
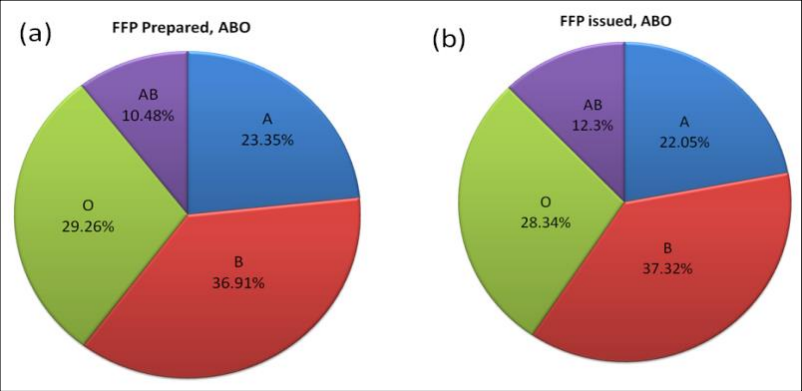
Frequency of ABO blood groups of FFP unit's prepared (c) and FFP units issued (d).

Compared to the ABO blood group frequency of blood donors (Figure 2(a)), a significantly (p = 0.0000) higher frequency of RDP units issued (Figure 4(b)) was observed in blood groups A (24.71%), B (39.34%), AB (11.52%), and a lower frequency of O (24.41%). Compared to the ABO blood group frequency of blood donors, there was a significantly (p = 0.00024) higher frequency of FFP units issued (Fig. 5(b)) in the blood group AB (12.3%) and a lower frequency of A (22.05%), B (37.32%), and O (28.42%). Compared to the ABO blood group frequency of blood donors, a significantly (p = 0.00081) higher frequency of RDP was prepared (Figure 4(a)) for blood groups A (24.18%) and AB (11.06%), whereas a lower frequency was observed for B (36.11%) and O (28.64%). Compared to the ABO blood group frequency of blood donors, FFP prepared (Figure 5(b)) was marginally (p = 0.65786) higher in blood group A (23.35%) and AB (10.48%) and marginally lower in B (36.91%) and O (29.26%).

Compared to the ABO blood group frequency of RDP units prepared, a significantly (p = 0.0000) higher frequency of RDP units issue (Table 1, Figure 4(a) & Figure 14(b)) was observed for blood group B (39.34%) and marginally higher for A (24.71%) and AB (11.53%), whereas a lower frequency of O (24.41%) was observed. Compared to the ABO blood group frequency of FFP units prepared (Table 1, Figure 5(a) & Figure 5(b)), a significantly (p = 0.01273) higher frequency of FFP units issued of blood group AB (12.3%) and B (37.32%) was observed, whereas a lower frequency of A (22.05%) and O (28.34%) was observed.

## Discussion:

The demographic pattern of our blood donors showed 99.11% were male and only 0.89% were female. The mean of the male donor's hemoglobin level was significantly higher (14.58 ± 1.58 gm/dl) than the female donor's (13.44 ± 0.85 gm/dl). The mean weight of male donors was also significantly higher (74.29 ± 11.73 kg) than that of female donors (65.54 ± 8.01 kg). Similar to our findings, study reports shows higher proportion of male blood donors in Coastal South India (95.2%), Brazil (99.6%), Saudi Arabia's Western Region (96.9%), Saudi Arabia's Central Region (82.98%), Ethiopia (86.8%), Cameroon (82.0%), and Nigeria (81.9%). This may be due to cultural stigma that blood donation of female donors may jeopardize their health. However almost equal proportion of both male and female blood donors had to be reported from; Belgium (54.6%), Spain (54.0%), United States (51.7%), Denmark (50.0%), France (50.0%), Netherlands (50.0%), United Kingdom (47.0%) and Finland (45.0%)[9].

The present study results shows, our blood donors have a higher frequency of B (37.53%) followed by O (29.29%) A (23.1%), and AB (10.09%), for ABO and a higher frequency of Rh(D)+Ve (95.19%) followed by Rh(D)-Ve (4.81%) for Rh blood group. The study reports of many authors, shows variations in blood group antigen expression can enhance or reduce the host's susceptibility to specific illnesses. Their study report shows, ABO and Rh blood group has association with specific disease susceptibility [8]. The association of blood groups with specific illnesses poses health risks. It has an impact on blood transfusion services by increasing or decreasing blood transfusions of the respective blood group compared to the blood group of the blood donor, which represents the blood group frequency of the general population.

Our study result shows that blood group A had a significantly higher frequency of 24.96% for blood issues (p = 0.0000) and 24.71% for platelet (RDP) issues (p = 0.0000) and a lower frequency of 22.05% for FFP issues (p = 0.00024) compared to the frequency of blood donors at 23.1%. The RBC surface of blood group A contains antigen A and the serum/plasma contains anti-B antibodies. Blood group A has been linked to a higher risk of HIV [10, 11], HBV [11], HCV [12], COVID-19 [13, 14], malaria, smallpox, enterotoxoid-mediated cholera, glue ear, capsular glaucoma, and heart disease, as well as a number of cancers, including gastric, breast, ovarian, cervical, leukemia (ALL), and leukemia of the pancreas[8], HCV related hepatocellular carcinoma (HCC)[15] Non-secretors of blood group A are linked to non-insulin-dependent diabetes, ankylosing spondylitis, Graves' disease, and coeliac disease [8].

Our study result shows that blood group B had a significantly higher frequency of 39.92% for blood issues (p = 0.0000) and 39.34% for platelet (RDP) issues (p = 0.0000) and a lower frequency of 37.32% for FFP issues (p = 0.00024), compared to the frequency of blood donors at 37.53%.The RBC surface of blood group B contains antigen B and the serum has anti-A antibodies. Blood group B has an association with a higher risk of HCV[12],Transfusion transmitted infections (TTIs) [112,^16^], COVID-19 [14], HCV related HCC [15], Malaria, Typhoid, Filariasis, Enterotoxoid-Mediated Cholera, Coeliac Disease, Ankylosing Spondylitis Graves disease and non-insulin dependent diabetes are associated with B non-secretors [8].

Our study result shows that blood group AB had a significantly higher frequency of 11.53% for platelet (RDP) issues (p = 0.0000), 12.32% for FFP issues (p = 0.00024), and a lower frequency of 9.76% for blood issues (p = 0.0000) compared to the frequency of blood donors at 10.09%. The RBC surface of blood group AB contains both A and B antigens and lacking anti-A or anti-B antibodies in their serum/plasma. Blood group AB has a higher risk of malaria [8].

Our study result shows that blood group O had significantly lower frequencies of 25.37% for blood issues (p = 0.0000), 24.41% for platelet (RDP) issues (p = 0.0000), and 28.34% for FFP issues (p = 0.00024) compared to the frequency of blood donors at 29.29%. The RBCs of blood group O have no antigens A and B on their surface and their serum/plasma has anti-A and anti-B antibodies. Blood group O has a higher risk of gastric ulcers, plague, a ruptured Achilles tendon, and parathyroid clear cell hyperplasia. Gastro-duodenal ulcers associated with blood group O non-secretors [9]. However, Blood group O has a lower risk of HIV, HBV [10, 11] HCV [12], COVID-19 infection [13,14], HCV related HCC [15] and TTIs [11,16].

Microbes and environmental materials that mimic ABO antigens have been demonstrated to activate naturally existing ABO system antibodies against ABO blood type antigens [8]. ABO antibodies are used by the body's innate immune system to target harmful bacteria and viruses that have ABO-active antigens. In addition, the innate immune response to an infection might differ based on blood group [8,17]. Blood groups, on the other hand, have the ability to act as phantom receptors. Bacteria, viruses, and parasites utilize certain blood groups as receptors and legends. For example, Plasmodium vivax and other malarial parasites, can bind to the Duffy blood group antigen [18,19]. Furthermore, the antigens found in specific blood types aid in membrane micro-domain retention, cell adsorption, and/or signal transmission. Variations in blood group antigen expression can enhance or reduce the host's susceptibility to specific illnesses [20].

Our study results show, blood group antigen expression may have an association with diseases and their severity. The susceptibility to various diseases or its severity may have an association with non-O blood groups (having antigens A, B, or both A and B but lacking A, B, or both A and B antibodies), causing a higher frequency of blood transfusions. On the other hand, the O blood group (lacking antigens A and B and having both A and B antibodies) may have a protective influence against diseases by their innate immune response, causing a lower frequency of blood transfusion compare to frequency of general population. More research is needed in this field to better understand the clinical relationship between antigen receptors and infection, specifically their etiology and relationship with blood group antigens and antibodies.

## Conclusion:

Compared to the ABO blood group frequency of blood donors, blood issues were higher in A and B and lower in O; platelet issues were higher in A, B and AB and lower in O; and FFP issues were higher in the AB. Blood group antigen expression and its susceptibility to various diseases or its severity may have an association with non-O blood groups, causing a higher frequency of blood transfusions. On the other hand, the O blood group may have a protective influence against diseases by their innate immune response, causing a lower frequency of blood transfusion.

## Funding:

This research did not receive any specific grants from funding agencies in the public, commercial, or not-for-profit sectors.

## Authors' contributions:

Sanjay Kumar Thakur, Anil Kumar Sinha, Dinesh Kumar Negi, and Sompal Singh prepared the study design. Sanjay Kumar Thakur performed the literature search and review, data collection, analysis of the data, and manuscript preparation. All the authors participated in data analysis, interpretation, and manuscript preparation. All authors have equally contributed to the preparation and critical review of the final version of the manuscript.

## Figures and Tables

**Table 1:** The blood group ABO and Rh (D) frequency and significance of comparative difference (χ²; P-value) between blood donors and blood units issued (whole blood and packed red cells), platelet (RDP) units issued, fresh frozen plasma (FFP) units issued, RDP units prepared, and FFP units prepared

	**A (%)**	**B (%)**	**AB (%)**	**O (%)**	**χ²; P-value**	**Rh(D)+Ve (%)**	**Rh(D)-Ve (%)**	**χ²; P-value**
Blood donors (n=23021)	5317 (23.1)	8639 (37.53)	2323 (10.09)	6742 (29.29)	-	21914 (95.19)	1107 (4.81)	
Blood units issued (n=20255)	5056 (24.96)	8085 (39.92)	1976 (9.76)	5138 (25.37)	93.085; 0	19254 (95.06)	1001 (4.94)	0.4134; 0.5202
RDP units issued (n=7239)	1789 (24.71)	2848 (39.34)	835 (11.53)	1767 (24.41)	68.4991;	-	-	-
FFP units issued (n=4164)	918 (22.05)	1554 -37.32	512 (12.3)	1180 (28.34)	19.2648; 0.00024	-	-	-
RDP units prepared (n=12211)	2953 (24.18)	4410 (36.11)	1351 (11.06)	3497 (28.64)	16.7051; 0.00081	-	-	-
FFP units prepared (n=7573)	1768 (23.35)	2795 (36.91)	794(10.48)	2216 (29.26)	1.60675; 0.65786	-	-	-
